# Predictors of premarital sexual initiation on adolescents in Bali: a longitudinal study

**DOI:** 10.1186/1471-2458-14-S1-O25

**Published:** 2014-01-29

**Authors:** Yuni Rahyani, Adi Utarini, Siswanto Agus Wilopo, Mohammad Hakimi

**Affiliations:** 1 Faculty of Medicine, Universitas Gadjah Mada (UGM), Yogyakarta 55281, Indonesia

## Background

One of the Millennium Development Goals (MDG’s) targets is improving reproductive and sexual health especially on adolescents. High-risk sexual behaviours like earliness of initiation of sexual intercourse in adolescents will have bad impact on their future. We need to explore the predictors of premarital sexual intercourse in adolescents based on theoretical framework. This study aimed to analyse predictors of premarital sexual intercourse initiation in adolescents based on Integrated Behavioural Model (IBM) framework, in order to make appropriate recommendations for policy and interventions.

## Materials and methods

A longitudinal study was carried out from October 2011 to March 2013, with three times follow-up of every six month. A total of 171 respondents at Grades 10 and 11 from two senior high schools in Denpasar city were included in this study. A survival analysis was used to analyse the data. Respondents fulfilled informed consent and self-reported questionnaire.

## Results

We found that no significant difference in premarital sexual intercourse initiation between male adolescents and female adolescents (p > 0.05). The median time of premarital sexual intercourse in male adolescents, who had lower intention to the initiation by Kaplan-Meier survival analysis, was shorter than adolescents who had higher intention. Female adolescents reported themselves as rape victims by their boyfriends. Female respondents with higher intention to initiation of premarital sexual intercourse had a 30% decrease in the opportunity to initiation of premarital sexual intercourse than respondents with lower intention (p > 0.05).

## Conclusions

We need to take a different approach for male and female adolescents, focusing on wider social determinants, and to increase the resiliency of adolescents. In addition there is a need to renew traditional interventions through the root of problems.

